# Beneficial Microorganisms Improve Agricultural Sustainability under Climatic Extremes

**DOI:** 10.3390/life13051102

**Published:** 2023-04-28

**Authors:** Arshad Jalal, Carlos Eduardo da Silva Oliveira, Poliana Aparecida Leonel Rosa, Fernando Shintate Galindo, Marcelo Carvalho Minhoto Teixeira Filho

**Affiliations:** 1 Department of Plant Health, Rural Engineering and Soils, Faculty of Engineering, São Paulo State University (UNESP), Av. Brasil 56—Centro, Ilha Solteira 15385-000, SP, Brazil; arshad.jalal@unesp.br (A.J.);; 2 Faculty of Agricultural Sciences and Technology, São Paulo State University (UNESP), Campus of Dracena, Sao Paulo 17900-000, SP, Brazil

**Keywords:** PGPBs, abiotic stresses, growth-promoting fungi, crop productivity, plant tolerance

## Abstract

The challenging alterations in climate in the last decades have had direct and indirect influences on biotic and abiotic stresses that have led to devastating implications on agricultural crop production and food security. Extreme environmental conditions, such as abiotic stresses, offer great opportunities to study the influence of different microorganisms in plant development and agricultural productivity. The focus of this review is to highlight the mechanisms of plant growth-promoting microorganisms (especially bacteria and fungi) adapted to environmental induced stresses such as drought, salinity, heavy metals, flooding, extreme temperatures, and intense light. The present state of knowledge focuses on the potential, prospective, and biotechnological approaches of plant growth-promoting bacteria and fungi to improve plant nutrition, physio-biochemical attributes, and the fitness of plants under environmental stresses. The current review focuses on the importance of the microbial community in improving sustainable crop production under changing climatic scenarios.

## 1. Introduction

**Figure 1:**
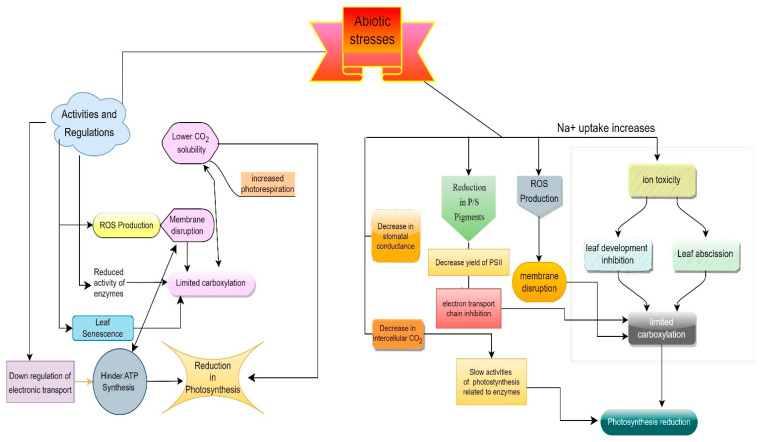
An overview of the effects of abiotic stresses on the different mechanisms of plants.

The severe impacts of transmutation with intense episodes of extreme weather can have significant consequences on agricultural outputs that should cause widespread food insecurity and affect survival of populations [1,2]. The severity, frequency, magnitude, and duration of extreme climatic events will become more highlighted and noticeable in the future [3]. The alterations in climate extremes have a direct or indirect influence on biotic and abiotic stresses with devastating impacts on agricultural crop production and food security [4]. Biotic stresses comprising phytopathogens and pests [5], as well as abiotic stresses including drought [6], soil salinity [6,7], heavy metals [8,9], flooding [10], high irradiance [11], low temperature [12] and high temperature [13], can cause intensified impacts on plant growth, physiology, metabolism, nutrient acquisition, and ecological desertification. The diverse effects of abiotic stresses on different mechanisms of plants are summarized in Figure 1.

In changing climate scenarios, intervention with microbes is considered a new sustainable strategy in agricultural production and mitigation of the resilient impacts of stresses [14]. The beneficial microbes and endophytes exhibit real-time amplifications to alleviate the devastating climatic impacts on plant health, physiology and biochemical aspects [14,15]. These microbial communities have several adaptations to abiotic stresses under different ecological processes, including facilitation of organic matter decomposition and nutrient acquisition in the rhizosphere of several plants [16]. Beneficial microbes, including plant growth-promoting rhizobacteria (PGPR), may have a controversial influence or no influence at all on plant growth and fitness under stressful environments, whereas other strains of PGPR have beneficial effects under climate-induced stressful extremes [17]. The PGPR engineered for agricultural practices boost plant growth, pathogen control, and microbial ecosystems by alleviating abiotic resiliencies [18,19].

Plant growth-promoting rhizobacteria tackle abiotic stresses by boosting several physiological and biochemical processes (nutrient uptake, photosynthesis, and source–sink relationships), metabolism and the regulation of homeostasis, osmotic potential, protein function, phytohormone production (indole-3-acetic acid and 1-aminocyclopropane-1-carboxylic acid deaminase), enzymatic activity, and nutrient solubilization [20,21,22]. To combat the punitive impact of abiotic stresses, numerous PGPR strains (including *Bradyrhizobium* sp. SUTNa-2 [23], *Pantoea dispersa* IAC-BECa-132, *Pseudomonas* sp., Enterobacter sp. [24], *Bacillus amyloliquefaciens* EPP90, *Bacillus subtilis*, *Bacillus pumilus* [25], *Curtobacterium* sp. SAK 1 [26], *Burkholderia phytofirmans* PsJNT [27], *Pseudomonas putida* KT2440 [28], *Enterobacter* sp. [29], *Serratia marcescens*, *Microbacterium arborescens*, *Enterobacter* sp. [30], *Bacillus cereus* PK6-15, *Bacillus subtilis* PK5-26 and *Bacillus circulans* PK3-109 [31], *Azospirillum lipoferum* FK1 [32], and *Azospirillum brasilense* Sp7 and *Azospirillum brasilense* Sp245 [33] have been used to facilitate the management mechanisms of different cereal and legume crops under stressful environments. Plant growth-promoting rhizobacteria employ various strategies to endure harsh weather conditions (Table 1).

**Figure 2:**
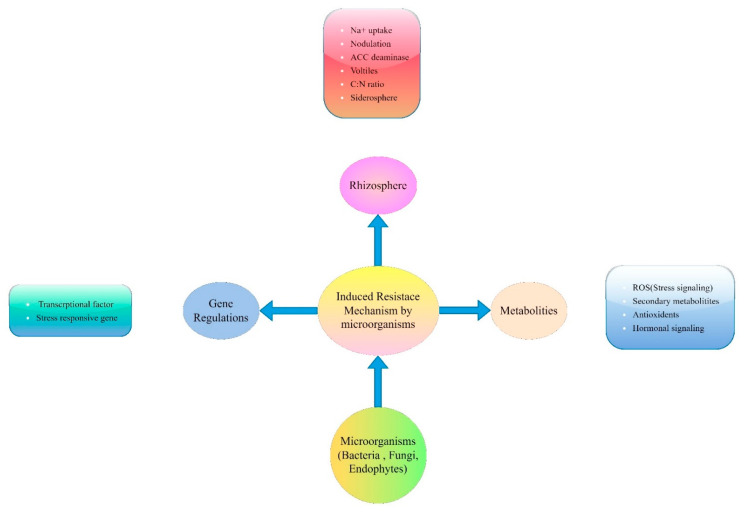
Mechanisms against abiotic stresses adapted from microorganisms.

In addition, root-associated microbes such as fungi can potentially influence different ecological processes to optimize plant health and growth, resulting in a great impact on plant physiology, nutrition, and survival ability that improves plant tolerance against environment-induced stresses [83]. These endophytic fungi confer abiotic stresses through the synthesis of various plant beneficial substances (ACC-deaminase, auxins, gibberellins, abscisic acid, siderophores) and solubilize nutrients for healthy plant growth [84,85]. The fugal endophytes form a mutualistic association with plants to promote photosystem activity, protein accumulation, primary metabolism that leads to higher growth, and tolerance under abiotic stresses [65,86]. Plants develop mutualistic relationships with several plant growth-promoting endophytic fungi, including *Piriformospora indica* [86], arbuscular Mycorrhizal fungi [65], *Trichoderma albolutescens*, *Trichoderma asperelloides*, *Trichoderma orientale*, *Trichoderma spirale*, and *Trichoderma tomentosum* [87], *Penicillium aurantiogriseum* 581PDA3, *Alternaria alternate* 581PDA5, *Trichoderma harzianum* 582PDA7 [88], and *Porostereum spadiceum* AGH786 [89], which can increase tolerance against abiotic stresses by improving the biochemical and physiological processes of different plants, as summarized in Figure 2.

The focus of this review is to highlight the mechanisms of plant growth-promoting microorganisms (especially bacteria and fungi) adapted to environmentally induced stresses such as drought, salinity, heavy metals, flooding, extreme temperatures, and intense light. The present state of knowledge focuses on the potential, prospective, and biotechnological approaches of plant growth-promoting bacteria and fungi to improve physiological and biochemical attributes and the fitness of plants under environmental stresses. Additionally, emphasis is placed on the significance of the role of microbial communities in promoting sustainable crop production amidst changing climatic scenarios.

## 2. Drought Stress

Disruption in the water cycle has become a serious challenge to overcome that is an alarming worry to farmers, horticulturists, and the world’s population as it threatens the food needs of humans and animals. In this context, farmers have increased the amount of irrigation to improve the quantity and quality of agricultural crops; however, this strategy could increase the cost of production [90]. Drought can be described as an unfavourable environmental condition with an insufficient level of moisture that can affect normal development and growth cycle of plants [91]. It has been highlighted that drought can reduce yield and cultivation potential (ideal yield) of soybean by up to 70% [92].

Severe climatic variations with unstable precipitation can result in prolonged drought in certain crops depending on the duration and intensity of drought [93], which ultimately affects crop development and productivity [94]. The effect of drought on yield is a highly complex mechanism that could adversely influence fertilization, embryogenesis, seed development, and the physiological, biochemical, and molecular processes of plants [95], which includes cell dehydration, reduced leaf size, stem elongation, root proliferation, nutrient uptake, and their use efficiency [96,97]. Drought also alters the signal activity of nitrogen and carbon metabolism enzymes, as well as the level of antioxidants in plants [98]. Plant signal genes are responsible for the accumulation of abscisic acid (ABA) via distinct regulatory pathways under drought stress conditions [99]. Modulation of gene expression related to drought stress is achieved by critical signaling pathways such as strigolactone, reactive oxygen species (ROS), and lipid-derived signaling [100,101]. Moreover, soluble sugar, programed cell death [99], and qualitative trait loci (QTL) [102] are gene expression adjustments in response to drought stress.

Alterations in the time and duration of precipitation generate long-term drought, which prominently affects the activities of microbial communities. The availability of water in the changing climate scenario is one of the most important factors that influences soil microbial activity [103]. Microbes adapt different strategies to deal with short- and long-term drought in response to changing climatic patterns [104]. Beneficial engineering of microorganisms within the root rhizosphere and root endosperm is a strategic approach to attaining healthy and productive crops under drought stress conditions [105]. Microbial communities under changing climatic conditions improve crop production efficiency [106]. Inoculation with microbes such as plant growth-promoting bacteria, fungi, and algae, either alone or in combination [107] is considered as one of the best alternatives to fertilizers that can enhance plant growth [108], root growth, and nutrient availability via mobilization and mineralization [109] and can help in the alleviation of drought stress [35]. These endophytic and epiphytic plant growth-promoting microbial diversities have adapted several mechanisms, such as synthesis of exopolysaccharide, 1-aminocyclopropane-1-carboxylate deaminase, volatile compounds, osmolytes, and antioxidants that can up- or downregulate stress-responsive genes, change root morphology, and improve nutrient uptake against drought stress in different cereal crops under changing climatic conditions [42,110]. Several plant growth-promoting microbes improve phosphorous and zinc solubilization, nitrogen fixation, and siderophore production and act as antimicrobial agents against harmful microbes that could reduce tolerance in food crops against drought stress and extreme climatic conditions [111,112].

Some beneficial fungi (arbuscular mycorrhizal fungi—AMF) and algae (*Amphora ovals*) adapt several biochemical, physiological, and molecular strategies to overcome drought conditions and improve crop growth and productivity under changing climate scenarios [113,114]. Plant growth-promoting fungi such AMF, Trichoderma spp., and certain algae promote antioxidant enzymes, nutrient uptake, chlorophyll, proline content, and phytohormone production, which can promote growth and tolerance against drought stress in host plants [113,115]. Over the last decade, many studies have demonstrated the use of plant growth-promoting bacteria and fungi that can mitigate the unfavourable effects of drought stress in host plants as summarized in Table 1.

## 3. Salt Stress

Salinity is one of the major global and environmental concerns that limits agricultural productivity and is attributed to extreme episodes of climatic changes [116]. Water quality and irrigation management irrespective of source, such as dams, ponds, rivers, artesian wells, or high-depth aquifers, contains salt complexes [117]. These salt complexes include some of the important cationic species, such as calcium (Ca^2+^), magnesium (Mg^2+^), sodium (Na^2+^), and potassium (K^+^), and among the anionic complexes are chloride (Cl^−^), carbonate (CO_3_^2−^), bicarbonate (HCO_3_^−^), sulfate (SO_4_^2−^), and boron (B) that all can have deleterious effects on agriculture ecosystems and plant productivity. Thus, the increased accumulation of these salts in low-quality irrigation water on arable land converts the land into non-usable and non-productive soil [118]. Soils irrigated with saturated water extract with an EC of 4.0 dS m^−1^ (40 mmol L^−1^ of NaCl) are considered to be saline and can cause osmotic pressure of 0.2 MPa that leads to a reduction in vegetable yields [119].

The expansion of salinity into formerly unaffected areas due to drastic climate changes can have adverse effects on plant growth through osmotic inhibition and phytotoxic effects on certain ions in the rhizosphere that trigger secondary oxidative stress in plants [116,120]. Salinity generates low water potential in the soil, thus restricting water availability for plants [121]. Plants with low osmotic potential under saline conditions often suffer from physiological drought that restricts nutrient mobilization to the aerial parts of plants. An excessive concentration of salt in the soil solution negatively affects plant physiology, photosynthesis, metabolism, protein and ATP synthesis, growth, and the productivity of crops [122]. The toxic effects of sodium (Na^+^) and chlorine (Cl^−^) ions are prevalent in saline soils, which disturbs enzymes and other macromolecules, thus damaging cellular organelles, disrupting photosynthesis and respiration, inhibiting protein synthesis, and causing ion-induced deficiencies [123].

Salinity negatively affects the photosynthetic rate of plants, which can impair crop productivity and cell membrane activity. Salinity also affects osmotic potential, which can reduce water availability, and further impacts CO_2_ permeability and deactivates the transport of photosynthetic electrons via shrinking intracellular spaces [124]. Stomatal closure can decrease carbon fixation and the production of reactive oxygen species (ROS) such as superoxide and single oxygen, which disrupt cellular processes by damaging lipids, proteins, and nucleic acids [125]. The unbalanced concentration of salt within the cell causes ionic toxicity and inhibits cell metabolism and other functional processes. Na^+^ can disrupt plant nutrition by inhibiting potassium ion (K^+^) uptake, which leads to the disturbance of enzymatic activity (K^+^ regulates more than 50 enzymes) within the cell [126]. The salt stress also triggers hormonal activity and alters assimilation and partition between sources and tissues [127]. Salinization alters phytohormones (abscisic acid, cytokinin, trans-Zeatin, indole-3-acetic acid, and carboxylic acid) in the tissues and nodules of the plant that cause leaf senescence and early tissue death [128]. It was demonstrated that carboxylic acid is the precursor of ethylene, which plays a vital role in the initiation of salt-induced senescence [129].

Plants adapt several strategies and evolutionary, physiological, and ecological processes to mitigate or tolerate salinity stress and improve productivity. The application of plant growth-promoting bacteria (PGPBs) is the most viable and effective alternative that can mitigate toxicity and the adverse effects of salinity while improving crop health and productivity [130]. These microorganisms mainly act as producers of phytohormones such as auxins, cytokinins, and gibberellins, which contribute to the growth of root systems, stimulate water absorption, and inhibit the effects of salinity [131,132]. Plant growth-promoting bacteria of different *Pseudomonas* sp. can improve peroxidase enzymes, total polyphenol and proline content, which are being indicated to increase relative water content in the leaves of *Coriandrum sativum* under salinity stress [133]. Plant prolines are the most adaptable and sensitive amino acids to stress conditions and can act as protectors of enzymes and defend plant tissues against osmotic stress [47].

The association of PGPBs with beneficial fungi has synergistic effects on plant growth through induced tolerance against saline conditions [134]. Arbuscular mycorrhizal fungi can improve crop performance and tolerance to salinization by reducing Na^+^ absorption while enhancing nutrient and water uptake and the antioxidant mechanisms of several plants [121,135]. Different species of ectomycorrhiza fungi (ECM), such as *Hebeloma*, *Laccaria*, *Paxillus*, *Pisolithus*, and *Rhizopogon*, can restrict Na+ transportation within plant tissues, thus improving mineral nutrition and water uptake and alleviating the effects of salination in host plants [136]. Trichoderma species are widely used as a biocontrol and plant growth-promoting agent in agriculture and can colonize in diverse substrates under different environmental conditions, therefore inducing tolerance against abiotic stresses [137].

Beneficial microorganisms are associated with increased water absorption, better use efficiency and uptake of nutrient, and improved soil fertility and structure, thus helping plants under salt stress conditions [138]. These microorganisms utilize nitrogen (N) for biological nitrogen fixation, nitrate reductase activity, and N use efficiency [139] while increasing phosphorous availability through phosphate solubilization [140]. In addition, these microorganisms can also increase the fertilizer use efficiency of NPK by 50% while alleviating the negative effects of salt stress in plants [141].

Over the past decade, numerous studies have highlighted the role of plant growth-promoting bacteria and fungi in mitigating the harmful effects of salt stress in plants (Table 1).

## 4. Heavy Metals

Heavy metals (HMs) are a serious threat to agriculture that can significantly harm different environmental, ecological, and nutritional factors of plants. The rising population has led to increased fertilizer use for higher food production, which can consequently lead to contamination of the environment and food chains [142]. The anthropogenic activities of humans, including mining, various industries, metallurgy, the use of chemical fertilizers containing HMs, and transportation, have led to a dramatic increase in HM accumulation in the ecosystem [143,144]. Heavy metals released into the air, environment, and soil can be absorbed by plants through roots and leaves, which can disrupt plant metabolism and cause several health risks to humans [143,145]. Edible plants are the major source of food in the human diet, and their contamination with toxic metals may result in catastrophic health hazards [143].

The term HMs refers to any metallic element that has a relatively high density and is either toxic or poisonous even at low concentration [142,143]. Heavy metals are generally categorized to belong to the group of metals and metalloids with high atomic density (density greater than 4 g cm^−3^) and mass [142]. Heavy metals include non-essential plant elements such as lead (Pb), cadmium (Cd), aluminum (Al), chromium (Cr), mercury (Hg), arsenic (As), silver (Ag), and platinum group elements [143,146]. Some heavy metals, such as copper (Cu), iron (Fe), manganese (Mn), zinc (Zn), nickel (Ni), and molybdenum (Mo), are essential micronutrients and are required for many of the biochemical functions of plants, including plant growth, oxidation and reduction reactions, electron transport, and many other metabolic processes; however, their high concentration can cause phytotoxicity [143,147].

Heavy metal toxicity in plants can cause leaf chlorosis, alter chlorophyll a and b ratios, decrease photosynthesis, inhibit root elongation, increase ROS production and membrane leakage, and change lipid composition through changing inter-cellular concentrations of nutrients [148,149].

Soils are a major sink for metal contamination in terrestrial ecosystems [131]. A diverse range of plants is used for the phytoremediation of toxic heavy metals and metalloids [150]. In addition, microorganisms such as PGPBs and PGPFs can enhance the effectiveness of phytoremediation [9,146,150] by producing organic acids, siderophores, bio-surfactants, bio-methylation, and redox processes that could transform heavy metals into soluble and bioavailable forms [9,150]. These microorganisms help the host plants by increasing biomass and phytoremediation attributes through synthesis of phytohormones such as indole-3-acetic acid (IAA) and enzyme like 1-aminocyclopropance-1-carboxylic acid deaminase (ACC), as well as through nitrogen fixation, P solubilization, and Fe sequestration [131,150]. These multiple traits improve the metabolic activity of microbes (Firmicutes, Proteobacteria, and Actinobacteria and most represented genera belong to *Bacillus*, *Pseudomonas*, and *Arthrobacter*) in heavy metal-contaminated sites [131,151].

Microbes play a key role in the remediation of HMs through phyto-stabilization, phyto-extraction, and phyto-volatilization [131,146]. Several studies have demonstrated the beneficial aspects of microbes in reducing HM toxicity in plant species over the past few decades (Table 2).

## 5. High Temperature

High temperature is one the major abiotic stress in extreme climates that has deleterious impacts on crop yield, global production, human health, and socio-economic damage and wildfires [173,174]. The exposure of plants to unsuitable temperatures during crop cycles results in reduced growth and biochemical aspects. Prolonged heat stress has severe implications on different metabolic processes, including water relations, heat shock proteins, carbohydrate metabolism, and physiological disruptions that lead to cell death [91,175]. High temperature stress crucially affects the grain filling stage [176], grain quality [177], grain protein content [178], biomass, phenology, leaf senescence, grain yield [179], and the plant canopy in wheat [180]. High temperature stress also has drastic influences on several crops, including rice [181], sorghum [182], pearl millet [183], maize [184], and wheat [185].

High temperature stress induces the production of reactive oxygen species (ROS), which damage the cell membranes of plants and trigger stress responses [186]. The ROS molecules encompass free radicals from oxygen (O_2_) metabolism, including superoxide radicals (O_2_^−^), hydroxyl radicals (OH^−^), hydrogen peroxide (H_2_O_2_), and singlet oxygen (1O_2_) [187]. Reactive oxygen species are produced via aerobic metabolism through the interaction of O_2_ and escaped electrons from electron transport chains in the chloroplast and mitochondria under normal conditions [188]. However, under stress conditions, accumulation of ROS affects cellular components and causes damage to membranes through lipid peroxidation [186,189]. Plants adapt several mechanisms, including the induction of antioxidants and signaling processes to overlap ROS damage [190] and the use of metabolites, proteins, and membrane lipids to cope with temperature stress [191].

Plant–microbial association (bacteria and fungi) is an alternative and climate resilient strategy that promotes plant growth and improves tolerance against abiotic stress [192], especially high levels of temperature stress [193]. These microorganisms fight against induced climatic changes (abiotic factors) that impair the general performance of plants by improving phytohormone synthesis, the availability of nutrients, water absorption, and structure, therefore contributing to the successful adaptation of plants under stressful conditions [138]. Beneficial microorganisms are involved in various mechanisms, such as the stimulation of phytohormones (indole-3-acetic acid (IAA), ethylene, cytokinins, gibberellins) [194], polyamines (speridine, spermine, cadaverine) [195], and solubilization of phosphate [196,197,198], and zinc [199,200,201], as well as production of secondary metabolites that can improve the stability of leaf cell membranes and leaf abscission, and plant tolerance to abiotic stresses [44,202].

In addition, these microorganisms may induce plant oxidative stress, reducing the deleterious effects of ROS [203]. Beneficial microorganisms such as bacteria, actinomycetes, and fungi provide shelter to host plants against extreme climatic events and unfavorable environmental alterations [204]. Several studies have highlighted the ameliorative effect of PGPBs [205,206] and PGPFs [65,115,207], which can increase tolerance against the negative impacts of high temperature stress in different crop plants. Furthermore, PGPBs and PGPFs can compensate and mitigate the adverse impact of high temperature, as is evident from the past twelve years of study (Table 3).

## 6. Low Temperature

Low temperature is also one of the most devastating environmental factors that affects plant growth and productivity. Occasional drops in the temperature of agricultural soils can affect the activity of terrestrial biota and plant growth. Low temperature corresponds to chilling (0–15 °C) that usually occurs in temperate regions and decreases plant productivity. These conditions stimulate the growth of saprophytic fungi that may disrupt soil nutrient cycling and compromise plant health [215]. Low temperatures disturb cellular homeostasis and some ROS, including hydrogen peroxide (H_2_O_2_), singlet oxygen (O_2_^−^), and HO^.^, and also disrupt some cellular functions related to proteins, lipids, carbohydrates, and DNA that may cause cell death in plants [217,227].

Several beneficial microorganisms have been reported to mitigate and alleviate the harsh impacts of abiotic stress, as indicated in Table 3. Different bacterial species, such as *Pseudomonas fragi*, *P. chloropaphis*, *P. fluorescens*, *P. proteolytica*, and *Brevibacterium frigoritolerans*, have been observed reducing freezing injuries and the content of lipid peroxides and ROS while stimulating some enzymatic activity (superoxide dismutase, catalase, peroxidase, and glutathione reductase) that could improve tolerance against cold stress in common bean seedlings [217]. Plant growth-promoting fungi such as *Trichoderma harzianum* and AMF (*Glomus mosseae*) are some of the most studied fungi in relation to improving resistance against cold stress conditions. These fungi could activate different enzymatic activity, discourage ROS production, and limit lipid peroxidation levels, which could decrease the damage caused by cold stress in tomato (*Solanum lycopersicum* L.) and *Elymus nutans* Griseb plants. 

## 7. Flood Stress and Oxygen Deficit

Global agriculture is severely affected by climate change. Flooding is one of the most drastic conditions of climate extremes and has detrimental impacts on soil fertility and nutrients, causing disruption to the crucial processes of plants [237]. The intensity and frequency of flooding is increasing due to climate extremes that could be a serious threat to the stability and productivity of ecosystems [238]. Plants frequently experience stresses that are typically caused by insufficient water or a lack of oxygen in flooding conditions. Flooding leads to localized depletion of oxygen due to stagnant water and sediment deposition on the soil surface [239]. The inhibition of cellular respiration and the submersion of non-photosynthetic plant tissues or roots under flooding are some of the most serious plant stresses [240].

Plants under flood stress undergo several physiological and molecular changes that might be due to the lack of oxygen availability affecting roots. Plants demonstrate certain symptoms under oxygen deficiency, such as the closing of stomata and a reduction in the water conductivity and growth of roots. Plants develop different morphological functions to cope with oxygen/flood stress, such as increases in gas diffusion in the roots, the accumulation of lignin and suberin at the cellular level, and the promotion of aerenchyma and adventitious roots [229]. Aerenchyma are specialized tissues that transport gases (O_2_) from aerial parts of the plant to the roots under oxygen deficit environments [240]. The aerenchyma are well developed in plants of aquatic and humid environments. Aerenchyma are developed in species of high economic importance, including plants such as sugarcane (*Saccharum* spp.), rice (*Oryza sativa*), barley (*Hordeum vulgare*), corn (*Zea mays*), wheat (*Triticum aestivum*), and soybeans (*Glycine max*) [240,241,242,243,244].

Plants undergo several metabolic alterations under flood stress, such as increased ethylene production and the signaling of stress hormones, which negatively interferes with plant morphology [222]. Flood stress causes anaerobic conditions that could reduce the microbial activity and enzymatic activity of plants in the rhizosphere [245]. Flood stress causes alterations in the structure of microbiota [246], which thus has consequences on the terrestrial biota and can enhance the role of bacteria and fungi in the decomposition of residues and nutrient cycling for the better performance of plants [247]. Understanding the behavior of potential soil microbiota in relation to flooding is one of the crucial discoveries that may confer stress tolerance in plants [240]. Several bacteria modulate the production of ethylene by plants through 1-aminocyclopropane-1-carboxylate (ACC) deaminase, which is the immediate precursor for ethylene synthesis. Plant growth-promoting bacteria reduce ethylene production, which can lead to the reduction of plant damage [248], as shown by Grichko and Glick [249] who reported that the inoculation of tomato (*Lycopersicon esculentum*) seeds with different bacterial strains (*Enterobacter cloacae* UW4, *E. cloacae* CAL2, and *Pseudomonas putida* ATCC17399/pRKACC or *P. putida* ATCC17399/pRK415) produced ACC deaminase. Plants at the vegetative growth stage were exposed to flooding stress for nine consecutive days, which produced AAC, chlorophyll a and b, and adventitious roots, as well as develop stem aerenchyma of the host plants to withstand under flood stress. Barnawal et al. [219] and Ravanbakhsh et al. [222] indicated that the inoculation of different plants with ACC deaminase-producing bacteria under flooded conditions increased plant growth by reducing ethylene production. The inoculation of *Cucumis sativus* with *Pseudomonas putida* UW4 under low available oxygen altered protein synthesis, nutritional metabolism, and antioxidant activity and promoted plant growth and defenses against stresses [220].

Beneficial microbes such as fungi prominently increase the tolerance of host plants under different environmental stresses [229]. Arbuscular mycorrhizal fungi applied to the roots of tomato plants under flooded and non-flooded conditions increased water relation and conductivity. It was also reported that indole-3-acetic acid (IAA) is one of the major phytohormones involved in the water conductivity of roots under low oxygen availability [229].

Several PGPBs and transgenic plants were studied under multiple stresses in field conditions. Farwell et al. [250] inoculated canola with *Pseudomonas putida* UW4 under nickel and flood stress and reported that *Pseudomonas putida* UW4 increased canola growth and biomass under flooding and heavy metal stresses. Cao et al. [239] indicated that flooding increased enzymatic activity in copper (Cu)-contaminated soil. In addition, the presence of Cu is inversely proportional to soil microbiota (bacteria and fungi), which could affect microbial communities and cause the immobilization of microelements under flooded and non-flooded conditions. The influence of beneficial microorganisms in improving tolerance to abiotic stresses (high and cold temperature and flooding) and regulating sustainable agricultural productivity under climatic extremes is summarized in Table 3.

## 8. Light Stress

Sunlight is one the major factors of photosynthesis that provides the necessary energy for plant growth and development. Despite this, intense light, especially its ultraviolet (UV) part, causes serious damage to DNA, proteins, and other cellular components of plants [251]. Sunlight damages photosynthetic machinery, primarily photosystem II (PSII), increases ROS production, and causes photo-inhibition that can hinder plant photosynthetic activity, growth, and productivity [252]. Excess light accelerates ROS production in PSI and PSII of chloroplasts, which may balance photo-inhibition and the repair of plant cells [252]. Light-triggered plant responses depend on the fluency, exposure time, and acclimation of plants before light exposure [251]. Reductions in the quantity and quality of light could signal plants to activate defensive systems by enhancing adaptive alterations in stem morphology [252]. The signaling pathways of light can balance the constructive and destructive impact of light on plant defense and growth mechanisms.

Microbes are less studied in the mitigation of light stress compared to other abiotic conditions. Some PGPBs have shown great potential by enhancing photosynthesis, chlorophyll content, and photosynthetic pigments that can reduce light damage [253]. The impact of light on the composition of rhizosphere communities, such as prokaryotes and fungi, can be increased or decreased under climatic extremes. There are several bacterial species, including *Pseudomonas* sp., *Massilia* sp., *Burkholderia* sp., and Acidobacteria, that are classified as beneficial microorganisms in the context of high light intensity. In addition, some fungal species, including *Geminibasidium* sp. and *Oidiodendron* sp., were also described as the most abundant species under intense light. The microorganism communities derived from soil under the influence of high light intensity are different in taxonomy and physiological characterizations. The impact of light on the soil rhizosphere includes the detection of *Pseudomonas* sp. that could consequently increase photosynthesis and carbon and nutrient assimilation [254]. Stefan et al. [255] verified that seed inoculation with *Bacillus pumilus* and *Bacillus mycoides* increased photosynthetic activity, water use efficiency, and chlorophyll content in runner bean (*Phaseolus coccineus* L.). Suzuki et al. [256] reported that *Acinetobacter calcoaceticus* could increase the chlorophyll content of lettuce (*Lactuca sativa* L.).

## 9. Conclusions

This review elaborated the importance of plant growth-promoting microorganisms (especially bacteria and fungi) that can mitigate the damage caused by environmentally induced stresses (drought, salinity, heavy metals, flooding, extreme temperatures, and intense light). This review determined the potential, prospective, and biotechnological approaches of plant growth-promoting bacteria and fungi for the alleviation of plants in response to environmental stresses. Some bacteria and fungi under abiotic stress conditions can improve physiological and biochemical processes, such as nutrient uptake, photosynthesis, source–sink relationships, metabolism and the regulation of homeostasis, osmotic potential, protein function, phytohormone production (indole-3-acetic acid and 1-aminocyclopropane-1-carboxylic acid deaminase), enzymatic activity, nutrient solubilization, and plant nutrition. Therefore, the use of plant growth-promoting bacteria (PGPBs) and fungi contributes positively to agricultural production in abiotic stress conditions.

Despite several studies demonstrating the benefits of beneficial microorganisms, there are still research gaps and restrictions on the molecular mechanisms of crops. A mechanistic understanding of the interactions of plants and microorganisms under abiotic stress should be developed to address agricultural difficulties and resolve the nutritional and production concerns that are brought by climatic extremes. Therefore, further studies involving microorganisms are recommended to enhance sustainable crop production and food security in the light of potentially unstable climatic conditions.

## Figures and Tables

**Table 1:** Summary of the positive effects of microbial agents in mitigating unfavorable drought and salt stress conditions in plants (2012–2020).

Microorganism	Stress	Plant Species	References
**Bacteria**			
*Azospirillum brasilense*	Drought	Marandu grass (*Urochloa brizantha*)	[34]
PGPRs strain IG 3, *Enterobacter ludwigii*, and *Flavobacterium* sp.	Drought	Wheat (*Triticum aestivum*)	[35]
*Bacillus* sp.	Drought	Sugarcane (*Saccharum* spp.)	[36]
*Bacillus megaterium*, *B. subtilis*, and *Bacillus thuringiensis*	Drought	Wheat (*Triticum aestivum* L.) and chickpea (*CicerArietinum*)	[37]
*Bacillus* sp. (12D6) and *Enterobacter* sp. (16i)	Drought	Wheat (*Triticum aestivum*) and maize (*Zea mays*)	[38]
*A* *ctinobacterium*	Drought	Maize (*Zea mays* L.)	[39]
*Proteobacteria*, *Actinobacteria*, *Gemmatimonadetes*, *Chloroflexi*, *Cyanobacteria*, and *Acidobacteria*	Drought	Cotton (*Gossypium hirsutum*)	[40]
*Bradyrhizobium japonicum* and *Azospirillum brasilense*	Drought	Soybean (*Glycine max*)	[41]
*Acinetobacter calcoaceticus EU- LRNA-72 and Penicillium* sp. *EU-FTF-6*	Drought	Foxtail millet (*Setaria italica* L.)	[42]
*Pseudomonas lini*, *Bacillus*, and *Serratia plymuthica*	Drought	Jujube (*Ziziphus jujuba*)	[43]
*Rhizobium tropici* and *Azospirillum brasilense*	Drought	Common bean (*Phaseolus vulgaris*)	[44]
*Azotobacter chroococcum*	Salt	Tomato (*Solanum lycopersicum*)	[45]
*Microbacterium oleivorans*, *Brevibacterium iodinum*, and *Rhizobium massiliae*	Salt	Pepper (*Capsicum annuum*)	[46]
*Bacillus* spp.	Salt	Pepper (*Capsicum annuum*)	[47]
*Pseudomonas* sp. and *Hartmannibacter diazotrophicus*	Salt	Alfalfa (*Medicago sativa*)	[48]
*Pantoea agglomerans*	Salt	Rice (*Oryza sativa*)	[49]
*Arthrobacter aurescens*, *A. woluwensis*, *Microbacterium oxydans*, *Bacillus megaterium*, and *B. aryabhattai*	Salt	Soybean (*Glycine max*)	[50]
*Bacillus aryabhattai* and *B. mesonae*	Salt	Tomato (*Solanum lycopersicum*)	[51]
*Pseudomonas* sp.	Salt	*Arabidopsis thaliana*	[52]
*Pseudomonas fluorescens*	Salt	Barley (*Hordeum vulgare*)	[53]
*Arthrobacter nitroguajacolicus*	Salt	Wheat (*Triticum aestivum*)	[54]
*Bacillus cereus* and *B. aerius*	Salt	Safflower (*Carthamus tinctorius*)	[55]
*Pseudomonas* and *Azospirillum brasilense*	Salt	Rapeseed (*Brassica napus*)	[56]
*Pseudomonas geniculate*	Salt	Maize (*Zea mays*)	[57]
*Bacillus halotolerans* and *Lelliottia amnigena*,	Salt	Wheat (*Triticum aestivum*)	[58]
**Fungi**			
*Glomus mosseae* and *Glomus intraradices*	Drought	Rose geranium (*Pelargonium graveolens* L.)	[59]
*Trichoderma atroviride* strain (TaID20G)	Drought	Maize (*Zea mays* L.)	[60]
*Gaeumannomyces cylindrosporus*	Drought	Maize (*Zea mays*)	[61]
Arbuscular mycorhizal fungi (AMF)	Drought	Sweet potato (*Ipomoea batatas* (L.) Lam.)	[62]
AM fungus *Funneliformis mosseae*	Drought	Trifoliate orange [*Poncirus trifoliata* (L.) Raf.]	[63]
*Trichoderma harzianum*	Drought	Tomato *(Solanum lycopersicum)*	[64]
*Rhizophagus intraradices, Funneliformis mosseae, F. geosporum*	Drought	Wheat (*Triticum aestivum*	[65]
*Arbuscular mycorrhizal fungi*	Drought	Chinese lyme grass (*Leymus chinensis*) and limpograss (*Hemarthria altissima*)	[66]
*Trichoderma harzinum 1*, *Trichoderma harzianum 2*, *Chaetomium globosum*, *and Talaromyces flavus*	Drought	Rice (*Oryza sativa* L.)	[67]
*Funneliformis mosseae, Glomus mosseae, G. intraradices*, *and G. etunicatum*	Salt	Desert grass (*Panicum turgidum*)	[68]
*Trichoderma harzianum*	Salt	Indian mustard (*Brassica juncea*)	[69]
*Trichoderma harzianum*	Salt	Tomato (*Solanum lycopersicum*)	[70]
*Trichoderma harzianum*	Salt	Rice (*Oryza sativa*) and maize (*Zea mays*)	[71]
*Klebsiella* sp.	Salt	Oat (*Avena sativa*)	[72]
*Glomus etunicatum*, *G. intraradices*, and *G. mosseae*	Salt	Cucumber (*Cucumis sativus*)	[73]
*Colobanthus quitensis* and *Deschampsia antarctica*	Salt	Lettuce (*Lactuca sativa*) and tomato (*Solanum lycopersicum*)	[74]
**Bacteria + Fungi**			
*Bacillus thuringiensis* + Arbuscular mycorrhizal fungus	Drought	French lavender (*Lavandula dentata*)	[75]
*Pseudomonas putida* + *Rhizophagus irregularis*	Drought	Calotrope (*Calotropis procera* Ait.)	[76]
*Micrococcus yunnanensis* + *Claroideoglomus etunicatum*	Drought	Moldavian balm *(Dracocephalum moldavica* L.)	[77]
*Pseudomonas fluorescens + Rhizophagus irregularis* or *Funneliformis mosseae*	Drought	Arizona cypress (*Cupressus arizonica* Green)	[78]
*Pseudomonas fluorescence* + *Glomus mosseae*	Salt	Bean (*Phaseolus vulgaris*)	[79]
*Methylobacterium oryzae* + *Glomus etunicatum*	Salt	Rice (*Oryza sativa*)	[80]
*Bacillus subitilis + Glomus. etunicatum*, *G. intraradices*, and *G. mosseae*	Salt	Acacia (*Acacia gerrardii*)	[81]
*Bradyrhizobium* sp. + *Trichoderma asperelloides*	Salt	Cowpea (*Vigna unguiculate*)	[82]

**Table 2:** Summary of the positive influence of microbes in mitigating heavy metal toxicity in contaminated sites (2010–2020).

Microorganism	Heavy Metal	Reference
**Bacteria**		
*Azotobacter chroococum* and *Rhizobium leguminosarum*	Pb	[152]
*Pseudomonas* sp. SRI2, *Psychrobacter* sp. SRS8, and *Bacillus* sp. SN9	Ni	[153]
*Sporosarcina ginsengisoli*	As (III)	[154]
*Bacillus cereus*	Cr (VI)	[154]
*P. macerans* NBRFT5, *B. endophyticus* NBRFT4, *B. pumilus* NBRFT9	Cu, Ni, and Zn	[155]
*Bacillus thuringiensis* GDB-1	As	[156]
*Bacillus cereus* strain XMCr-6	Cr (VI)	[157]
*Bacillus subtilis*	Cr (VI)	[158]
*Pseudomonas putida*	Cr (VI)	[158]
*Pseudomonas* sp. LK9	Cd, Cu, and Zn	[159]
*Enterobacter* sp. And *Klebsiella* sp.	Cd, Pb, and Zn	[160]
*Kocuria flava*	Cu	[154]
*Pseudomonas veronii*	Cd, Cu, and Zn	[154]
*Bacillus pumilus* E2S2 and *Bacillus* sp. E1S2	Cd and Zn	[161]
*Enterobacter cloacae* B2-DHA	Cr (VI)	[162]
*Planomicrobium chinense*, *B. cereus*, *P. fluorescens*	Co, Mn, Ni, and Pb	[163]
*B. cereus*, *P. moraviensis*	Mn and Cd	[164]
*B. safensis* FO-036b (T) and *P. fluorescens*	Pb and Zn	[165]
**Fungi**		
*Pleurotus platypus*	Ag	[166]
*Rhizopus oryzae* (MPRO)	Cr (VI)	[167]
*Aspergillus versicolor*	Cu and Ni	[154]
*Aspergillus fumigatus*	Pb	[168]
*Rhizopus oryzae*	Cu	[169]
**Algae**		
*Spirogyra* spp. and *Cladophora* spp.	Cu (II) and Pb (II)	[154]
*Spirogyra* spp. and *Spirullina* spp.	Cr Cu, Fe, Mn, and Zn	[154,170]
*Cystoseira barbata*	Cd, Ni, and Pb	[171]
*Hydrodictylon*, *Oedogonium*, and *Rhizoclonium* spp.	As	[172]

**Table 3:** Summary of the positive effects of microbes in mitigating unfavorable high and cold temperature and flooding stress conditions in plants (2012–2020).

Microorganism	Stress	Plant Species	Reference
**Bacteria**			
*Azospirillum brasilense* and *Bacillus amyloliquefaciens*	High temperature	Wheat (*Triticum aestivum*)	[175]
*Bacillus amyloliquefaciens*	High temperature	Rice (*Oryza sativa*)	[205]
*Bacillus amyloliquefaciens*	High temperature	Wheat (*Triticum aestivum*)	[208]
*Pseudomonas syringae*	High temperature	*Arabidopsis thaliana*	[209]
*Enterobacter* sp.	High temperature	*Arabidopsis thaliana*	[210]
*Bacillus velezensis*	High temperature	Wheat (*Triticum aestivum*)	[211]
*Bacillus cereus*	High temperature	Tomato (*Solanum lycopersicum*)	[212]
*Bacillus cereus*	High temperature	Tomato (*Solanum lycopersicum*)	[213]
*Pseudomonas*, *Bacillus*, *Stenotrophomonas*, *Methylobacterium*, *Arthrobacter*, *Pantoea*, *Achromobacter*, *Acinetobacter*, *Exiguobacterium and Staphylococcus*, *Enterobacter*, *Providencia*, *Klebsiella and Leclercia*, *Brevundimonas*, *Flavobacterium*, *Kocuria*, *Kluyvera*, and *Planococcus*	Cold temperature	Wheat (*Triticum aestivum*)	[214]
*Arthrobacter*, *Flavimonas*, *Flavobacterium*, *Massilia*, *Pedobacter*, and *Pseudomonas*	Cold temperature	Tomato (*Solanum lycopersicum*)	[215]
Rhizobacterial isolates of *Bacillus* genera, Gu2 and 127b	Cold temperature	Wheat (*Triticum aestivum*)	[216]
*Pseudomonas fragi*, *P. chloropaphis*, *P. fluorescens*, *P. proteolytica*, and *Brevibacterium frigoritolerans*	Cold temperature	Bean (*Phaseolus vulgaris* L.)	[217]
*Bradyrhizobium japonicum*	Flooding	Soybean (*Glycine max*)	[218]
*Achromobacter xylosoxidans*, *Serratia ureilytica*, *Herbaspirillum seropedicae*, and *Ochrobactrum rhizosphaerae*	Flooding	Tulsi (*Ocimum sanctum*)	[219]
*Pseudomonas putida*	Flooding	Cucumber (*Cucumis sativus*)	[220]
*Azospira oryzae*, *Pelomonas saccharophila*, and *Methylosinus* sp.	Flooding	Rice (*Oryza sativa*)	[221]
*Pseudomonas putida*	Flooding	*Rumex palustris*	[222]
**Fungi**			
*Glomus deserticola* and *Glomus constrictum*	High temperature	Tomato (*Solanum lycopersicum*)	[223]
*Aspergillus japonicas*	High temperature	Soybean (*Glycine max*) and sunflower (*Helianthus annuus*)	[224]
*Thermomyces* sp.	High temperature	Cucumber (*Cucumis sativus*)	[225]
*Thermomyces lanuginosus*	High temperature	*Cullen plicata*	[226]
*Glomus mosseae*	Cold	*Elymus nutans* Griseb	[227]
*Trichoderma harzianum*	Cold	Tomato (*Solanum lycopersicum* L.)	[115]
*Glomus versiforme and Rhizophagus irregularis*	Cold	Barley (*Hordeum vulgare* L.)	[228]
*Rhizophagus irregularis*	Cold	Cucumber (*Cucumis sativus* L.)	[15]
*Rhizophagus irregularis*	Flooding	Tomato (*Solanum lycopersicum*)	[229]
*Glomus intraradices*, *G. versiforme*, and *G. etunicatum*	Flooding	Cattail (*Typha orientalis*) and rice (*Oryza sativa*)	[230]
*Trichoderma*	Flooding	Rice (*Oryza sativa*)	[231]
*Aspergillus fumigatus*	Flooding	*Arabidopsis* sp.	[232]
Bacteria and fungi			
*Bradyrhizobium* + arbuscular mycorrhizal fungi	High temperature	Soybean (*Glycine max* L.)	[233]
Proteobacteria, Actinobacteria, Chloroflexi, and Nitrospirae + Dothideomycetes, Sordariomycetes, and Ascomycota	High temperature and drought	Sorghum (*Sorghum bicolor* L.) and foxtail millet (*Setaria italica* L.)	[234]
*Bacillus* and *Pseudomonas* + *Penicillium*	Cold temperature	Potato (*Solanum tuberosum*)	[235]
*Paraburkholderia graminis* C4D1M and *Funneliformis mosseae*	Cold temperature	Tomato (*Solanum lycopersicum* L.)	[236]

## Data Availability

Not applicable.
